# Structure of AI Responses With Complex Patient Analysis for Patient Alternatives and Patient Modifiers

**DOI:** 10.1111/eje.70108

**Published:** 2026-02-13

**Authors:** David C Johnsen, Leonardo Marchini, Kim Vo, L Brendan Young, Shareef M Dabdoub

**Affiliations:** ^1^ Pediatric Dentistry, College of Dentistry University of Iowa Iowa City Iowa USA; ^2^ Preventive and Community Dentistry, College of Dentistry University of Iowa Iowa City Iowa USA; ^3^ Second Year Dental Student, College of Dentistry University of Iowa Iowa City Iowa USA; ^4^ Biostatistics and Computational Biology, College of Dentistry University of Iowa Iowa City Iowa USA

**Keywords:** AI patient alternatives, AI structure analysis, critical thinking

## Abstract

**Background:**

Artificial intelligence (AI) is already a powerful tool that is rapidly growing within the dental sector. Reports of structure and characteristics of AI responses to patient scenarios are limited.

**Purpose:**

To analyse characteristics and structure of AI responses to prompts for treatment planning alternatives and patient alternatives for complex patients. The project is seen as a prelude to exploring the interface between AI information and responsibility for patient decisions, patient privacy and credibility of AI information.

**Methods:**

Microsoft Copilot from July 2025 was prompted for a hypothetical patient scenario to develop treatment alternatives. In addition to treatment alternatives, patient analysis factors were prompted and three were analysed in the manuscript: patient modifying factors, patient capacity to subscribe to professional recommendations and prognoses.

**Results:**

Qualitative analyses for AI responses were extensive, in categories, amenable to table format, informational and not recommendational. For each treatment alternative AI generated goal, phases, pros and cons. AI offered ten modifying factors affecting dental treatment, including age, medical conditions, medications, etc. For patient capacity, AI generated seven responses under headings of positive indicators and limitations. For prognoses, AI generated short‐and long‐term prognoses with key indicators. Treatment alternatives remained largely unchanged before and after sequential inclusion of patient modifying factors.

**Conclusions:**

AI can offer extensive categorised information on patient care to reinforce the dentist of considerations in patient care without making recommendations. Responsibility for patient decisions, patient privacy and soundness of information remains with the dentist.

## 1 Introduction

Artificial intelligence (AI) is already a powerful tool and continues to advance in its use, especially in dentistry. While AI can assist the clinician and dental educator in making decisions, final responsibility for patient care rests with the clinician. ‘Responsibility’ has several components: patient care decision‐making, patient privacy, credibility of AI information [1, 2, 3]. A first step to articulate the interface between AI information and responsibility for the dentist can be to analyse and describe the structure and characteristics for AI responses to patient care scenarios. Discussion has centred around general AI actions more than explicit structure and characteristics for AI responses. There has been less discussion centred around how and how much AI can assist the dentist in making reasonable decisions and presenting viable treatment alternatives [1, 2, 3, 4, 5].

The interface between AI and dentist responsibility with patients can follow clarification for structure of AI responses. An ongoing discussion revolves around the interface where AI leaves off and where responsibility takes over. Does the clinician need to know and understand more or less about patient care and more or less about learning than before AI? [6, 7, 8] A follow up purpose from the present project will be to explore the interface between AI and responsibility—scenarios where AI can play a more prominent role leading closer to responsibility and where AI has some of the same uncertainties that the clinician has.

The AI‐responsibility interface has limited literature. For example, a recent report by the Veteran's Administration on AI in Dentistry offers an extensive review of areas where AI has been used to analyse different aspects of dentistry, with examples for radiology, diagnostic tools, workflow optimisation, efficiencies in patient flow, etc. [9, 10, 11] The matter of responsibility is implied with statements like, ‘…Its clinical usefulness lies in synergizing technological capabilities with the irreplaceable value of human diagnostics and clinician‐patient relationship’ [6]. Still largely missing from the discussion are explicit depictions of AI functioning in a patient case setting—how and how much the intervention brings the dentist or educator closer to confidence in assuming decision and care responsibility.

The purposes of this project are to analyse characteristics and structure of AI responses to patient scenario type of prompts to understand: (1) What is the interface between AI information and the point of responsibility for decisions to maximise AI effectiveness? And (2) What are characteristics and what is the structure of AI responses to prompts regarding assessment and planning for complex patients. The hypotheses for AI's contribution would be that AI prompts on a specific case scenario will offer an explicit depiction of alternatives for the oral health aspects and patient characteristics (non‐procedural) of care.

## 2 Methods

The project started out to explore the characteristics and content of responses to AI prompts on treatment planning for complex patients. Little literature exists on the characteristics and content of AI responses calling for treatment plans for complex patients. The initial route chosen was to use a patient profile constructed from several real patients used in a class for predoctoral students. The reasoning was that this approach would challenge the AI with a complex patient situation and would obviate clearance from IRB. We had no basis to anticipate the resulting characteristics or content of AI responses to prompts. Prompts were based on questions from published patient‐based, student‐led demonstrations of thinking and judgement used in class. As soon as we saw AI responses to prompts, we could for the first time see a ‘shape’ and content of AI responses to prompts calling for treatment options for a complex patient. The process of analysis was thus retroactive and not proactive since we could not design an analysis for something for which we could not anticipate the characteristics and content. Two authors assembled the AI responses to prompts on treatment planning. Three authors reviewed the AI responses to prompts. A process of progressive consensus with iterations of exchange was used by three authors to offer the responses in the tables. By consensus, the same three authors developed a set of patterns for responses shown in the tables. Exact wording from AI responses to prompts was used in condensing responses into patterns. The process is descriptive and exploratory based on pattern recognition.

To test AI responses, Microsoft Copilot as of July 2025 was prompted for a hypothetical patient scenario to develop treatment alternatives. Microsoft Copilot was used in this study due to HIPAA‐compliant systems. One potential outcome with this approach is a more explicit depiction of AI input with a specific case than would be possible from more abstract discussion about how AI can be used in patient analysis. This approach is particularly appropriate because treatment planning resulting in viable alternatives is at the heart of critical thinking in dental education and is vital for the practicing dentist [12, 13, 14]. Critical thinking in patient care challenges both students and professionals to weigh research‐based evidence, consider patient‐specific factors and anticipate outcomes—skills that are essential in clinical decision‐making [15]. Exploring how AI can contribute to these complex tasks provides a meaningful test of its potential to support—not replace—professional judgement and responsibility. The application of this line of thinking is thus applicable to the practicing dentist and the dental educator.

### 2.1 For the Section on Treatment Alternatives From a Hypothetical Patient/Case

A synthesised patient case used for a student exercise served as a prompt to Microsoft Copilot. The case material does not represent any one real patient (See Addendum for the complete case scenario used for the prompt.) Microsoft Copilot was initially prompted with the case information followed by the prompt: ‘Considering the attached dental patient scenario, please prepare 3 dental treatment options’.

The outcome of the initial, direct prompt asking for three treatment alternatives was compared to the results of a series of incremental prompts with a more nuanced sequence of clinical decision‐making steps. For this comparison output, Microsoft Copilot was prompted sequentially for each of the following steps emulating the thought process of the expert as previously reported [16, 17, 18].

- Prompt 2.1: List modifying factors and how dental treatment is affected by each modifying factor.
- Prompt 2.2: Which data are most important for treatment planning? Why?
- Prompt 2.3: Prioritise the patient's conditions.
- Prompt 2.4: What if we do nothing? Six months? Five years?
- Prompt 2.5: What is the patient's capacity to subscribe to professional recommendations?
- Prompt 2.6: What is the patient's risk and disease progression? Show four choices from ROHD.
- Prompt 2.7: What are treatment alternatives?
- Prompt 2.8: What are the short‐ and long‐term prognoses?
- Prompt 2.9: What are recommendations to maintain health?

For each sequential prompt, Microsoft Copilot was prompted to consider each of the previous questions. For example, for the prompt on prognoses, the following was entered:

Prompt #2.8.: ‘Considering the same attached dental patient scenario and your response to the previous 7 questions, what are the short‐ and long‐term prognoses for each treatment alternative?’

For planning of care for the hypothetical patient, there was no anticipation of the quality and quantity of AI responses; the anticipation was to look for patterns of AI responses and seek consensus to describe the patterns. For each call for AI responses for the complex patient/case, the following issues were examined: extent of response; categorisation of responses; amenability to table format for easier visual analysis; spectrum of responses from informational to recommendational. The importance of each of these categories is thought to be of enough importance to report on each of these items when analysing each factor.

## 3 Results

### 3.1 Responses to Prompts for Treatment Alternatives

Qualitative results are shown starting with the responses for prompts calling for treatment alternatives. From the list of responses to the structured prompt, sample responses are shown. Microsoft Copilot responses to each of the prompts for the sequence of clinical decision‐making steps resulted in extensive information ranging from more prescriptive interventions to more precautionary things to keep in mind. Three samples are then shown: Responses to Prompt #2.1 on patient modifying factors, responses to Prompt #2.5 on patient's capacity to subscribe to professional recommendations, and responses to Prompt #2.8 on short‐ and long‐term prognoses.

### 3.2 AI Responses to the Original Prompt for Treatment Alternatives

Responses were extensive, responses were in categories, responses were amenable to table format and responses were informational and not recommendational.

### 3.3 AI Responses for Patient Modifying Factors

These responses are sequential to the direct prompts for treatment alternatives. Again, responses were extensive, responses were in categories, responses were amenable to table format and responses were informational and not recommendational.

The sequence of prompts for Prompt 2.1 on patient modifiers resulted in treatment options almost identical to the treatment options in Table 1. Responses to patient modifiers for Prompt 2.1 are extensive, are notably different from the listing of treatment options, and are listed in Table 2. A preliminary assessment of patterns by the authors for descriptions of responses indicated that responses were extensive/insightful/informational/educational. Responses listed ten patient characteristics, each with commentary of the impact and treatment considerations. Responses for treatment options were in the language/context of the oral cavity. Responses to prompts on patient modifying factors were focused more on the person. The two lists are seen as distinctly different with little overlap and are seen as instructional for the dentist. While the responses are extensive, the Microsoft Copilot does not cross the line of responsibility. The dentist is to make the final decision on which patient modifying factors are most important for patient decision‐making. Responses are regarded by the authors as more informational/cautionary than listings for treatment options.

**TABLE 1:** Short‐ and long‐term prognoses for each dental treatment.

Option	Name	Goal	Phases/Treatment plan	Pros	Cons
1	Phased Removable Partial Denture (RPD) Rehabilitation	Restore function and aesthetics gradually, with affordability in mind	Phase 1: Disease control (interim restorations, extractions, hygiene) Phase 2: Interim RPDs Phase 3: Definitive RPDs	Cost‐effective Flexible timeline Improves chewing & aesthetics Minimally invasive	Multiple appointments May be less comfortable than fixed options
2	Selective Fixed Restorations + RPD	Preserve strategic teeth with crowns and combine with RPDs	Crown restorable teeth (e.g., #29, #31) Extract non‐restorable teeth Fabricate RPDs using crowned teeth as abutments	Improved RPD retention Better prognosis for strategic teeth	Higher cost than Option 1 Still requires removable prosthesis More complex planning
3	Minimal Intervention + Monitoring	Prioritise comfort, hygiene and minimal intervention due to medical/financial factors	Extract only symptomatic or severely decayed teeth Temporary/glass ionomer restorations Emphasise preventive care Delay prosthetics	Least expensive Least invasive Prioritises patient comfort	Doesn't restore full function/aesthetics Risk of further deterioration without follow‐up

**TABLE 2:** Modifying factors affecting dental treatment planning.

#	Modifying factor	Impact	Treatment consideration
1	Age (71 years old)	Slower healing, reduced salivary flow, higher systemic risk	Use conservative, minimally invasive treatments; avoid overly complex or lengthy procedures
2	Medical conditions		
	Hypertension (150/92)	Risk during invasive procedures	Monitor vitals; minimise or cautiously use epinephrine
	Hypercholesterolemia	Possible cardiovascular risk	Consider medical clearance before surgery
	Parkinson's disease	Motor control issues affect hygiene and cooperation	Schedule visits around medication timing; use hygiene aids
	Anxiety	Affects compliance and comfort	Create calm environment; use shorter appointments
3	Medications		
	Buspirone (Dry mouth)	Increases caries risk, discomfort	Use fluoride, saliva substitutes; increase recall frequency
	Carbidopa/Levodopa (Vomiting)	Risk of enamel erosion, aspiration	Position upright; monitor nausea
	Polypharmacy	Drug interaction risks	Review meds thoroughly before prescribing
4	Allergies (Penicillin, Sulfa)	Limits antibiotic choices	Use alternatives like clindamycin or azithromycin
5	Mobility Issues (Uses walker)	Difficulty accessing office/chair	Ensure accessibility; allow more time per visit
6	Financial constraints	Limits treatment options and timeline	Phase treatment; prioritise urgent care; use cost‐effective options
7	Lack of dental insurance	May delay or limit care	Offer clear cost breakdowns; refer to low‐cost clinics or dental schools
8	Poor oral hygiene & gingivitis	Increased periodontal risk	Emphasise prevention, instruction, regular cleanings
9	Xerostomia (Dry mouth)	Caries risk; affects denture retention	Recommend saliva substitutes, fluoride, xylitol
10	Complex dental history	History of extractions, RCTs, crowns	Consider long‐term prognosis; plan prosthetics accordingly

### 3.4 AI Responses to Patient Capacity to Subscribe to Professional Recommendations

These responses are sequential to the direct prompts for treatment alternatives.

Responses to Prompt #2.5 on patient capacity to subscribe to professional recommendations also show treatment options similar to prompt #1 calling for treatment options without considering the sequence of clinical decision‐making steps. Responses to the prompt on patient capacity to respond to professional recommendations are seen as extensive, informational and precautionary things to keep in mind. Copilot has created three categories for consideration not called for in the original prompt on the vertical axis of Table 3, with each one instructional for items for the dentist to consider. On the horizontal axis of Table 3 are details and implications for each category of capacity to subscribe to professional recommendations. Responses are regarded by the authors as insightful/informational. Responses include factors to keep in mind with commentary on each factor as shown in Table 3. Responses to the capacity to subscribe to professional recommendations are seen as more informational/precautionary than are responses for treatment options. Responses in the Microsoft Copilot‐created categories of ‘Positive indicators for Capacity’ and ‘Modifying factors that may limit capacity’ have an array of responses and are seen as more explicit than responses for the Copilot‐created category of ‘Treatment Planning implications’. For example, a response as a positive indication of capacity is ‘Engaged in Care’ with the implication ‘shows willingness to participate and follow through the care’; this is seen as an observation to proceed, less explicit than responses for treatment options and more explicit than responses under ‘Treatment planning implications’. A comparative example under ‘Treatment planning implications’, a response is ‘Emphasise preventive care and hygiene support’ and is followed by a ‘Detail’ of ‘Mitigate future issues with manageable interventions’; the last comments are seen as ambiguous. Microsoft Copilot does not cross the line to assume responsibility for the options offered.

**TABLE 3:** Assessment of patient capacity to follow professional recommendations.

Category	Details	Implications for treatment
**Positive indicators of capacity**		
Cognitive function appears intact	No signs of cognitive decline; patient seeks and consents to treatment	Patient can understand and engage with treatment plans
Motivated by function	Expressed desire to improve chewing and oral function	Likely to comply if treatment goals align with functional improvements
Engaged in care	Returned for care voluntarily despite no insurance; underwent comprehensive exam	Shows willingness to participate and follow through with care
**Modifying factors that may limit capacity**		
Financial constraints	No insurance; prefers phased/affordable care	Need for staged treatment; prioritise urgent and cost‐effective procedures
Medical and physical limitations	Parkinson's disease affects dexterity; uses walker	Schedule short appointments; ensure accessibility and comfort
Dry mouth & limited hygiene effectiveness	Brushes once daily; shows gingivitis and plaque	Require strong preventive focus; consider caregiver involvement if available
**Treatment planning implications**		
Use phased, flexible treatment plans	Adapt to finances and tolerance	
Emphasise preventive care and hygiene support	Mitigate future issues with manageable interventions	
Use clear verbal and written instructions	Reinforce understanding and compliance	
Schedule short, well‐timed appointments	Align with Parkinson's medication peak for best cooperation	

### 3.5 AI Responses for Prognoses

These responses are sequential to the direct prompts for treatment alternatives. Responses to Prompt #2.8 on short‐ and long‐term prognoses for each treatment alternative again show treatment options similar to prompt #1 calling for treatment options without the sequence of clinical decision‐making steps. Responses to the prompt on prognoses had consensus of the authors as extensive, informational and precautionary things to keep in mind as shown in Table 4. Copilot has created categories for consideration, with each one instructional for items for the dentist to consider; each of the created categories are seen as a useful set of reinforcers for the dentist in managing the person as well as the dentition. Responses are regarded by the authors as insightful/informational. Responses include each treatment ‘option’, ‘prognoses’ for short‐ and long‐term and ‘Key Considerations’. Responses have an extensive narrative and are seen as informational/precautionary. For example, following the heading ‘treatment option’ of ‘Phased RPD Rehabilitation’ which is explicit, under ‘Key considerations’, is the response ‘Long‐term success depends on hygiene, follow‐up and completing definitive prostheses’. For each of the ‘treatment options’, responses for ‘key considerations’ are seen as less explicit than the respective ‘treatment options’. The range of unpredictability for ‘Key considerations’ is seen as similar to the range of unpredictability for the dentist without AI.

**TABLE 4:** Sustained Caries Prevention Strategies for High‐Risk Populations.

Treatment option	Short‐term prognosis	Long‐term prognosis	Key considerations
1. Phased RPD Rehabilitation	Fair to Good	Fair to Good	Can be done in phases, supporting affordability
			Interim RPDs restore function early
			Long‐term success depends on hygiene, follow‐up and completing definitive prostheses
2. Fixed Restorations + RPD	Guarded to Fair	Good (if completed)	Higher upfront cost and complexity
			Requires multiple appointments and patient endurance
			Offers better prosthesis stability and retention long‐term
3. Minimal Intervention + Monitoring	Good (for comfort and stabilisation)	Poor to Guarded	Prioritises comfort and affordability
			Does not restore full function or aesthetics
			High risk of further deterioration if not followed up properly

## 4 Discussion

AI is already a powerful tool to bring the dentist closer to the moment of responsibility for decisions on patient care and on learning guidance and performance assessment. A qualitative analysis of the structure and characteristics for AI responses is seen as a prelude to engaging the interface between AI information and responsibility, in this case for patient alternatives. AI responses fulfilled each of the criteria outlined in Methods: for patient alternatives, responses were extensive, responses were in categories, responses were amenable to table format, and responses were informational without being recommendational for each of the prompts. For patient alternatives, the significance is that the dentist is provided with a series of insightful considerations for the patient without AI making explicit recommendations. Responses were seen as instructional for the dentist and for the student. Responses for treatment alternatives were more procedural technically; responses for patient characteristics, capacity to subscribe to professional recommendations and prognoses centred more on the person and less on the dentition. Responses to treatment alternatives compared to patient modifying factors are seen as complimentary. To gain a view of the dentition as well as the person, it is up to the dentist or dental educator to target an array of prompts to gain an overall view of the patient and their oral conditions.

The amount of relevant information provided is remarkable. AI not only offers specific input for a single patient/case but also offers a more general outline for protocols for patient care. The dentist or dental educator are still in the position of determining which data are more important and why. Whether analysing data acquired from a patient interview and exam or analysing AI responses, reliability of the data is still a responsibility of the dentist or dental educator. It is worth noting that AI did not attempt to take responsibility in any of the responses. While it is speculation on what assuming responsibility would look like, one option could be, ‘Procedure X is the decision for this patient’.

The purposes of the project are seen as largely successful. The structure and characteristics called for with AI responses were fulfilled with examples shown: extensive, in categories, amenable to table format, informational, not recommendational. The potential outcome expressed in the initial articulation of purpose is seen as largely fulfilled: the graphic expression of what AI can do with a specific patient/case would likely not be possible with an abstract commentary of what AI can do.

For patient treatment options, a consistent pattern was seen with explicit procedural options and less explicit options for long term patient behaviour guidance such as capacity to subscribe to recommendations and prognoses (Table 5). Thus, AI and the dentist/educator are similar in that both have more definitive procedural actions and less definitive actions to guide personal behaviours. Stated differently, AI may help the dentist more quickly to procedural options and is less help in guiding long term behaviours essential for sustained health and minimum disease. This is not surprising as AI large language models, such as Microsoft Copilot, are based on existing data, and it has been reported that data is sparse on effectively guiding long term behaviours for sustained health and minimum disease, especially among high‐risk and vulnerable populations with recurring disease [19].

**TABLE 5:** Summary of CoPilot Responses to treatment options, modifying patient factors, Capacity to follow recommendations and Prognoses for treatment options (will eliminate 5 & 6 on interventions).

Summary of AI‐generated dental decision support outputs		
Prompt	AI output summary	Key implications
1. Treatment options	Three options proposed: (1) Phased RPD Rehabilitation; (2) Strategic Fixed Restorations + RPD; (3) Minimal Intervention + Monitoring.	AI clearly delineated procedural alternatives with pros/cons; strongest on structured, procedural care.
2. Modifying patient factors	Age, comorbidities (Parkinson's, hypertension), medications, xerostomia, anxiety, financial constraints, mobility, prior dental history.	AI appropriately adjusted treatment planning per risk factors; emphasised phased care and accessibility.
3. Capacity to follow recommendations	Patient motivated and cognitively intact; constrained by finances, hygiene challenges and physical limitations.	AI recognised both facilitators and barriers to care compliance; reinforced need for flexible, simplified plans.
4. Prognosis by treatment option	Option 1: Fair‐to‐good short/long‐term; Option 2: Guarded short‐term, good if completed; Option 3: Good short‐term, poor long‐term.	AI effectively projected realistic outcomes; relied on treatment feasibility, maintenance and patient support.

The findings have implications for the dental educator attempting to guide learning with the same thought processes as the practicing dentist. In addition to the explicit options, the extra‐oral factors displayed offer a powerful set of reinforcers for the dentist to consider even though there are no explicit recommendations. It is pointed out that Microsoft Copilot did not interact with the patient. ‘Reading the patient’ would likely influence the dentist's view on capacity to subscribe to professional recommendations and prognoses, for better or worse. Factors with little emphasis in AI responses are the person's capacity to subscribe to professional recommendations (willing/able?) and domestic stability (stable/not stable?).

An issue for discussion is the amount of data generated from the series of prompts for patient care. Responses are extensive; a question may arise whether the extensive responses are practical in a busy practice. While there is no certainty on how much is enough information, there is general agreement on getting to know key factors about the patient. The responses from (eight) different perspectives on the person beyond the oral cavity are seen as having potential value in organising the dentist‐patient interactions; in some ways the responses are a summary of a course on perspectives in dentist‐patient interactions.

Implications for both the practitioner and educator seem considerable. Both will be using AI, and both will have colleagues or students using AI. So, some familiarity seems logical on what AI responses will look like in patient assessment. A further implication is the volume of data gathered for the practicing dentist or for the student, also on time constraints, deciding on what is important and why. Dealing with data overload from AI and other sources is a skill for the practitioner and student for continued growth.

AI is here to stay. The key is to analyse where it can aid the dentist and dental educator in taking responsibility for decisions and where AI leaves off with human judgement as the final decision‐maker. It does seem likely that with AI, the dentist and dental educator will need the same command of their field as with pre‐AI. These findings highlight not only where AI can support decision‐making but also where it falls short in the same places that challenge even the most experienced clinicians and educators. Guiding long‐term health behaviours, understanding a patient's capacity to follow recommendations, and adapting to complex psychosocial contexts remain deeply human challenges that AI cannot yet resolve. The interface between AI and human responsibility is not a handoff but a collaboration as AI continues to stay and evolve.

## 5 Limitations and Future Projects

The project with extensive AI responses was then intentionally limited to the exploration of AI information on patient alternatives to Copilot. Future projects can expand to include other AI platforms. An inherent limitation is whether the authors were astute enough to develop the most insightful prompts. Future projects can explore additional questions. With these limitations, this project may offer a baseline for articulating one structure and set of characteristics for AI information for alternatives for care of complex patients.

## Funding

The authors have nothing to report.

## Conflicts of Interest

The authors declare no conflicts of interest.

## Data Availability

All data are available from the present study.

## Appendix 1 Case Information

Patient is a 71‐year‐old female from a nearby town. She lives at home with her husband. Pt walks with a walker. The patient does not have dental insurance and is very money conscious. She wants to space out her treatment over several months because of her limited finances.

### Health History

- Previous c section.
- Hypertension (BP: 150/92).
- Hypercholesterolemia.
- Parkinson's disease.
- Anxiety—this occurs when she gets stressed, has never had a problem in the dental chair.
- Allergies to penicillin and sulfa drugs.

#### Medications

**Table:**

Drug	Indication	Oral Side Effect
Amantadine	Parkinsons	
Buspirone	Anxiety	Occasional dry mouth
Carbidopa/Levadopa	Parkinsons	Vomiting
Lovastatin	Cholesterol	
Metoprolol	Hypertension	
Montelukast	Seasonal allergies	

#### Dental History

Patient came in for a dental exam in 2015 and at that time she had dental insurance. Her teeth do not cause her pain or sensitivity. At the time of her exam, she had 4 teeth with previous RCT and 5 teeth with full coverage crowns which she says were done years ago. Four teeth had previously been extracted for various reasons. Pt wants to improve her chewing capabilities. At the exam she was diagnosed with caries on 5 teeth and treatment planned for an URPD/LRPD (See radiographs).

##### Treatment Provided in 2015

#5—carious pulp exposure, patient elected for extraction.

#9—composite restoration.

#19—extracted.

#20—glass ionomer restoration.

#30—carious pulp exposure, patient elected for extraction.

Master impressions for URPD/LRPD—at this appointment, pt was notified that she lost dental insurance coverage and opted not to pursue the RPDs at this time.

##### Limited Treatment in 2016 and 2017

2016—#3 was extracted because it was non‐restorable.

2017—#18 was extracted because it was non‐restorable.

2018—Patient came for a dental appointment because she broke tooth #25. The tooth does not cause her pain. Pt elected to have a comprehensive exam.

Below are the teeth present in the mouth and carious surfaces for each tooth.

#### Exam Findings

Below are the teeth present in the mouth and carious surfaces for each tooth.

**Table:**

			DIF	M (i)	I	MID	D	M	DIF		M	
			#6	#7	#8	#9	#10	#11	#12		#15	
#31	#29	#28	#27	#26	#25	#24	#23	#22	#21	#20		#17
MB	DFL	DF	MDF	MDFL	RT	MDFL	MDFL	MLD	DF	DF		Bony

#17 complete bony impaction, can't see this tooth from the mouth.

#29 previous RCT and PFM crown.

#31 previous RCT and gold crown.

Probing depths are 3 mm or less.

Generalised gingivitis.

Generalised plaque accumulation along the gingiva and interproximal.

Pt says her mouth feels dry and she sips on water throughout the day.

Pt says she brushes with a manual toothbrush once daily.
